# Crystal structure of chlorido­(2-{[2-(4-chloro­phen­yl)hydrazin-1-yl­idene-κ*N* ^1^](phen­yl)meth­yl}pyridine-κ*N*)(η^5^-penta­methyl­cyclo­penta­dien­yl)iridium(III) tetra­phenyl­borate

**DOI:** 10.1107/S2056989015003023

**Published:** 2015-02-18

**Authors:** Neelakandan Devika, Nandhagopal Raja, Subbiah Ananthalakshmi, Bruno Therrien

**Affiliations:** a Department of Chemistry, BIT Campus, Anna University, Tiruchirappalli 620 024, Tamil Nadu State, India; b Institut de Chimie, Université de Neuchâtel, Avenue de Bellevaux 51, CH-2000 Neuchâtel, Switzerland; c Department of Chemistry, Urumu Dhanalakshmi College, Tiruchirappalli 620 019, Tamil Nadu State, India

**Keywords:** crystal structure, hydrazinyl­idene­pyridine ligand, iridium(III) complex, penta­methyl­cyclo­penta­dien­yl, intra­molecular C—H⋯Cl hydrogen bond, C—Cl⋯π inter­actions, C—H⋯π inter­actions

## Abstract

The title compound, [Ir(η^5^-C_5_Me_5_)Cl(C_18_H_14_ClN_3_)]B(C_6_H_5_)_4_, is chiral at the metal center and crystallizes as a racemate. In the cation, the hydrazinyl­idene­pyridine ligand is *N*,*N*-coordinated through the *N*-pyridyl and *N*-hydrazinyl­idene groups forming a five-membered metallacycle. An intra­molecular C—H⋯Cl hydrogen bond is observed. In the crystal, centrosymmetrically-related cations are connected by C—Cl⋯π inter­actions, forming a dimeric structure. The crystal packing is further stabilized by weak inter­ionic C—H⋯π inter­actions.

## Related literature   

For the pharmacological and catalytic properties of penta­methyl­cyclo­penta­dienyl iridium complexes, see: Johnpeter *et al.* (2013 ▸); Liu & Sadler (2014 ▸); Raja & Therrien (2014 ▸). For background to the chemistry and properties of hydrazinyl­idene­pyridine derivatives, see: Liu *et al.* (2002 ▸); Ghedini *et al.* (2004 ▸); Marandi *et al.* (2015 ▸); Devika *et al.* (2015 ▸); Ghosh *et al.* (2011 ▸, 2012 ▸). For the structures of related compounds, see: Prasad *et al.* (2010 ▸); Payne *et al.* (2013 ▸).
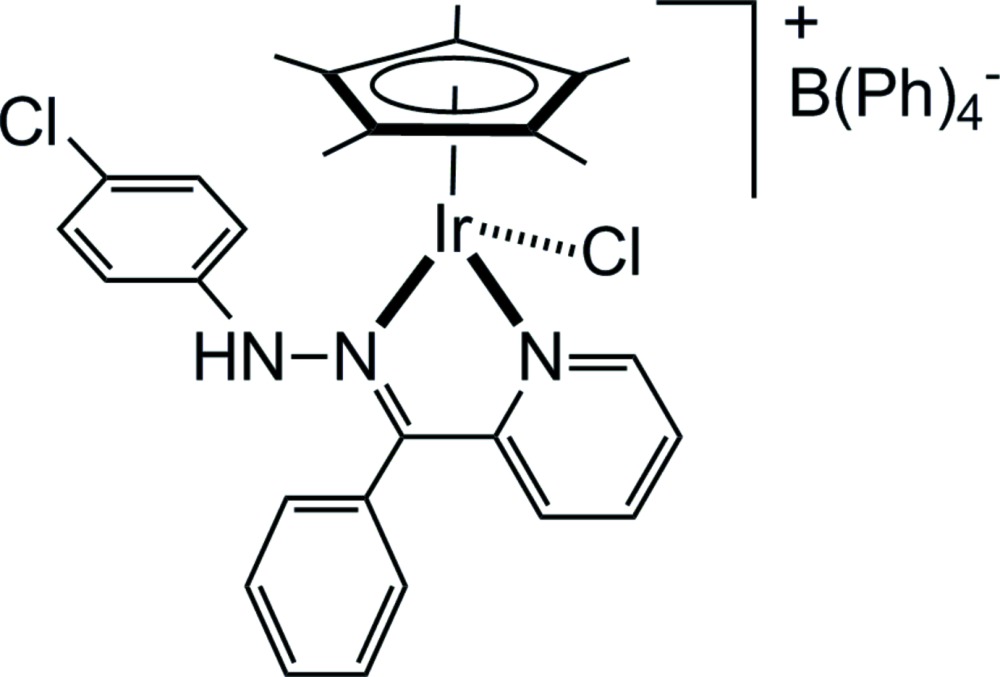


## Experimental   

### Crystal data  

[Ir(C_10_H_15_)Cl(C_18_H_14_ClN_3_)](C_24_H_20_B)
*M* *_r_* = 989.85
Triclinic, 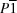

*a* = 8.9597 (4) Å
*b* = 12.5586 (6) Å
*c* = 20.0796 (9) Å
α = 98.228 (4)°
β = 95.860 (4)°
γ = 97.183 (4)°
*V* = 2201.95 (17) Å^3^
*Z* = 2
Mo *K*α radiation
μ = 3.19 mm^−1^
*T* = 173 K
0.23 × 0.19 × 0.18 mm

### Data collection  

Stoe IPDS diffractometer
Absorption correction: empirical (using intensity measurements) (*DIFABS*; Walker & Stuart, 1983 ▸) *T*_min_ = 0.261, *T*_max_ = 0.715
43057 measured reflections
11895 independent reflections
10423 reflections with *I* > 2σ(*I*)
*R*_int_ = 0.067

### Refinement  

*R*[*F* ^2^ > 2σ(*F* ^2^)] = 0.026
*wR*(*F* ^2^) = 0.058
*S* = 0.95
11895 reflections
541 parameters
H atoms treated by a mixture of independent and constrained refinement
Δρ_max_ = 1.58 e Å^−3^
Δρ_min_ = −1.54 e Å^−3^

### 

Data collection: *IPDS* *EXPOSE* (Stoe & Cie, 2000 ▸); cell refinement: *IPDS* *CELL* (Stoe & Cie, 2000 ▸); data reduction: *IPDS* *INTEGRATE* (Stoe & Cie, 2000 ▸); program(s) used to solve structure: *SHELXS97* (Sheldrick, 2008 ▸); program(s) used to refine structure: *SHELXL97* (Sheldrick, 2008 ▸); molecular graphics: *ORTEP-3* for Windows (Farrugia, 2012 ▸); software used to prepare material for publication: *SHELXL97*.

## Supplementary Material

Crystal structure: contains datablock(s) I, global. DOI: 10.1107/S2056989015003023/rz5149sup1.cif

Structure factors: contains datablock(s) I. DOI: 10.1107/S2056989015003023/rz5149Isup2.hkl

**Fig. 1.**
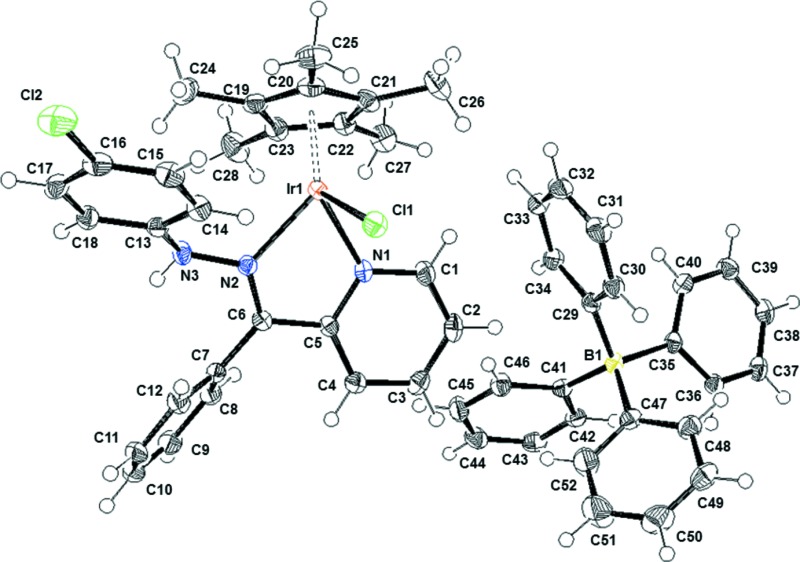
The molecular structure of the title compound with displacement ellipsoids drawn at the 50% probability level.

. DOI: 10.1107/S2056989015003023/rz5149fig1.tif
The mol­ecular structure of the title compound with displacement ellipsoids drawn at the 50% probability level.

**Fig. 2.**
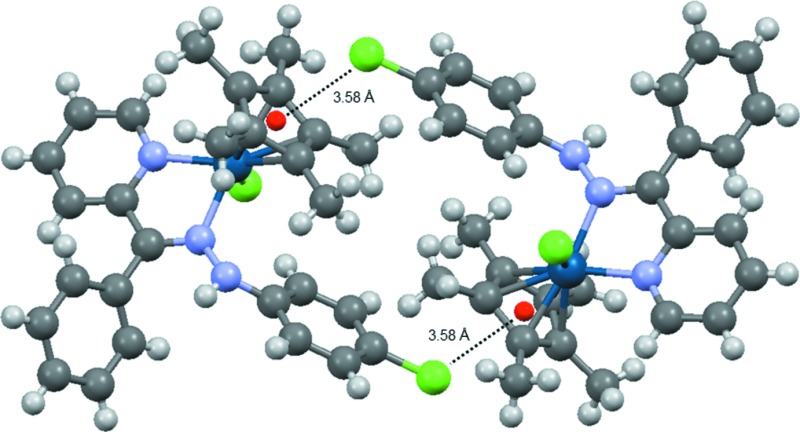
Dimeric structure involving two centrosymmetrically related cations.

. DOI: 10.1107/S2056989015003023/rz5149fig2.tif
Dimeric structure involving two centrosymmetrically related cations.

CCDC reference: 1048992

Additional supporting information:  crystallographic information; 3D view; checkCIF report

## Figures and Tables

**Table 1:** Hydrogen-bond geometry (, ) *Cg*1, *Cg*2, *Cg*3, *Cg*4 and *Cg*5 are the centroids of the C29C34, C41C46, C35C40, C7C12 and C19C23 rings, respectively.

*D*H*A*	*D*H	H*A*	*D* *A*	*D*H*A*
C14H14Cl1	0.93	2.64	3.553(3)	168
C2H2*Cg*1	0.93	2.71	3.463(3)	139
C11H11*Cg*2^i^	0.93	2.61	3.365(3)	139
C18H18*Cg*3^ii^	0.93	2.60	3.518(3)	169
C37H37*Cg*4^iii^	0.93	2.65	3.489(3)	150
C16Cl2*Cg*5^iv^	1.75(1)	3.58(1)	4.359(3)	105(1)

Symmetry codes: (i) 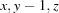; (ii) 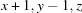; (iii) 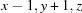; (iv) 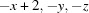.

## supplementary crystallographic information

### S1. Chemical context

In recent years, penta­methyl­cyclo­penta­dienyl iridium complexes have shown great promises as anti­cancer agents (Johnpeter *et al.*, 2013; Liu & Sadler, 2014). Moreover, penta­methyl­cyclo­penta­dienyl iridium complexes are well known for their catalytic activity (Raja & Therrien, 2014). Among ligands to coordinate to a penta­methyl­cyclo­penta­dienyl iridium core, hydrazinyl­idene­pyridine derivatives are quite attractive (Liu *et al.*, 2002; Ghedini *et al.*, 2004; Marandi *et al.*, 2015). These non-symmetrical *N*,*N*-bidentate ligands introduce chirality at the metal center (Devika *et al.*, 2015), and they are also known to be biologically relevant molecules (Ghosh *et al.*, 2011, 2012).

### S2. Structural commentary

Herein, we present the synthesis and characterization of a chiral-at-metal penta­methyl­cyclo­penta­dienyl iridium(III) hydrazinyl­idene­pyridine complex, [Ir(η^5^-C_5_Me_5_)Cl(C_18_H_14_ClN_3_)]B(C_6_H_5_)_4_. The molecular structure is shown in Figure 1. The cationic complex adopts a typical piano-stool geometry and it is chiral at the metal center. The salt crystallizes as a racemate in the triclinic space group *P*-1. In the cationic complex, the hydrazinyl­idene­pyridine ligand is *N*,*N*-coordinated, the *N*-hydrazinyl­idene and the *N*-pyridyl groups forming with the iridium center a five-membered metallacycle. Upon coordination, the hydrazinyl­idene­pyridine ligand is non-planar, an angle of 55.15 (6)° is observed between the planes formed by the pyridyl and {(4-chloro­phenyl)­hydrazinylene}methyl groups. Otherwise, all geometrical data around the iridium(III) center are similar to those found in related *N*,*N*-chelated penta­methyl­cyclo­penta­dienyl iridium complexes (Prasad *et al.*, 2010; Payne *et al.*, 2013). An intra­molecular C—H···Cl hydrogen bond is present (Table 1).

### S3. Supra­molecular features

In the crystal packing of the title compound, centrosymmetrically-related cations form through the penta­methyl­cyclo­penta­dienyl and 4-chloro­phenyl groups a dimeric structure, the chlorine atoms sitting above the centroids of the C_5_Me_5_ rings at 3.58Å (Fig. 2). In addition, crystal packing is stabilized by weak inter­ionic C—H···π inter­actions (Table 1).

### S4. Synthesis and crystallization

The title compound was synthesized by reacting one equivalent of (η^5^-C_5_Me_5_)_2_Ir_2_(µ-Cl)_2_Cl_2_ (100 mg, 0.126 mmol) with two equivalents of 2-{1-[2-(4-chloro­phenyl)­hydrazin-1-yl­idene](phenyl)­methyl}­pyridine (Marandi *et al.*, 2015) (77 mg, 0.25 mmol) in methanol (25ml), and the mixture was refluxed for 6 hours. To the hot solution was added sodium tetra­phenyl­borate (92 mg, 0.25 mmol). Then a reddish brown precipitate was observed, and after filtration, the solid was purified by column chromatography (silica gel, chloro­form:methanol, 9.9:0.1 *v*/*v*). Crystals suitable for a single-crystal X-ray structure analysis were obtained by slow evaporation of a di­chloro­methane/*n*-pentane (1:1 *v*/*v*) solution of the title compound. Yield: 60%. IR (KBr, ν, cm^-1^): 1590 (s, C═N). ^1^H NMR (400 MHz, CD_3_CN, 25°C): δ (ppm) = 8.80 (d, ^3^J_H—H_ = 5.6 Hz, 1H, H_ar_), 8.00 (dd, ^3^J_H—H_ = 8.4 Hz, 1H, H_ar_), 7.79 (dd, ^3^J_H—H_ = 7.2 Hz, 1H, H_ar_), 7.54 (m, 10H, H_ar_), 7.27 (m, 8H, H_B(Ph)4_), 7.10 (br s, 1H, NH), 6.99 (dd, ^3^J_H—H_ = 7.2 Hz, 8H, H_B(Ph)4_), 6.84 (dd, ^3^J_H—H_ = 7.2 Hz, 4H, H_B(Ph)4_), 1.43 (s, 15H, C_5_Me_5_). MS (ESI positive mode): m/z 670.0 [M - B(Ph)_4_]^+^.

### S5. Refinement

Except for the amine H atom, which was located in a difference Fourier map and refined freely, all hydrogen atoms were included in calculated positions and treated as riding atoms, using SHELXL-97 default parameters, with C–H = 0.93Å for C_arom_ and 0.96Å for CH_3_, with U_iso_(H) = 1.2 U_eq_(C) or 1.5 U_eq_(C) for methyl H atoms.

### Crystal data

**Table:**

[Ir(C_10_H_15_)Cl(C_18_H_14_ClN_3_)](C_24_H_20_B)	*Z* = 2
*M**_r_* = 989.85	*F*(000) = 996
Triclinic, *P*1	*D*_x_ = 1.493 Mg m^−^^3^
Hall symbol: -P 1	Mo *K*α radiation, λ = 0.71073 Å
*a* = 8.9597 (4) Å	Cell parameters from 8000 reflections
*b* = 12.5586 (6) Å	θ = 1.9–28.9°
*c* = 20.0796 (9) Å	µ = 3.19 mm^−^^1^
α = 98.228 (4)°	*T* = 173 K
β = 95.860 (4)°	Block, red
γ = 97.183 (4)°	0.23 × 0.19 × 0.18 mm
*V* = 2201.95 (17) Å^3^	

### Data collection

**Table:**

Stoe IPDS diffractometer	11895 independent reflections
Radiation source: fine-focus sealed tube	10423 reflections with *I* > 2σ(*I*)
Graphite monochromator	*R*_int_ = 0.067
phi oscillation scans	θ_max_ = 29.2°, θ_min_ = 1.7°
Absorption correction: empirical (using intensity measurements) (*DIFABS*; Walker & Stuart, 1983)	*h* = −12→11
*T*_min_ = 0.261, *T*_max_ = 0.715	*k* = −17→17
43057 measured reflections	*l* = −27→27

### Refinement

**Table:**

Refinement on *F*^2^	Primary atom site location: structure-invariant direct methods
Least-squares matrix: full	Secondary atom site location: difference Fourier map
*R*[*F*^2^ > 2σ(*F*^2^)] = 0.026	Hydrogen site location: inferred from neighbouring sites
*wR*(*F*^2^) = 0.058	H atoms treated by a mixture of independent and constrained refinement
*S* = 0.95	*w* = 1/[σ^2^(*F*_o_^2^) + (0.0305*P*)^2^] where *P* = (*F*_o_^2^ + 2*F*_c_^2^)/3
11895 reflections	(Δ/σ)_max_ = 0.002
541 parameters	Δρ_max_ = 1.58 e Å^−^^3^
0 restraints	Δρ_min_ = −1.54 e Å^−^^3^

### Special details

**Table:**

Experimental. A crystal was mounted at 173 K on a Stoe Image Plate Diffraction System (Stoe & Cie, 2000) using Mo *K*α graphite monochromated radiation. Image plate distance 100 mm, φ oscillation scans 0 - 180°, step Δφ = 1.2°, 5 minutes per frame.
Geometry. All e.s.d.'s (except the e.s.d. in the dihedral angle between two l.s. planes) are estimated using the full covariance matrix. The cell e.s.d.'s are taken into account individually in the estimation of e.s.d.'s in distances, angles and torsion angles; correlations between e.s.d.'s in cell parameters are only used when they are defined by crystal symmetry. An approximate (isotropic) treatment of cell e.s.d.'s is used for estimating e.s.d.'s involving l.s. planes.
Refinement. Refinement of *F*^2^ against ALL reflections. The weighted *R*-factor *wR* and goodness of fit *S* are based on *F*^2^, conventional *R*-factors *R* are based on *F*, with *F* set to zero for negative *F*^2^. The threshold expression of *F*^2^ > σ(*F*^2^) is used only for calculating *R*-factors(gt) *etc*. and is not relevant to the choice of reflections for refinement. *R*-factors based on *F*^2^ are statistically about twice as large as those based on *F*, and *R*- factors based on ALL data will be even larger.

### Fractional atomic coordinates and isotropic or equivalent isotropic displacement parameters (Å^2^)

**Table:**

	*x*	*y*	*z*	*U*_iso_*/*U*_eq_	
C29	0.3816 (3)	0.68256 (18)	0.26342 (12)	0.0194 (5)	
C30	0.2854 (3)	0.6179 (2)	0.20884 (13)	0.0246 (5)	
H30	0.1834	0.6003	0.2136	0.029*	
C31	0.3370 (4)	0.5790 (2)	0.14788 (14)	0.0307 (6)	
H31	0.2698	0.5359	0.1132	0.037*	
C32	0.4876 (4)	0.6044 (2)	0.13898 (14)	0.0322 (6)	
H32	0.5220	0.5792	0.0983	0.039*	
C33	0.5866 (3)	0.6679 (2)	0.19136 (15)	0.0291 (6)	
H33	0.6882	0.6854	0.1860	0.035*	
C34	0.5338 (3)	0.7053 (2)	0.25201 (14)	0.0231 (5)	
H34	0.6024	0.7474	0.2866	0.028*	
C41	0.4432 (3)	0.75292 (19)	0.39947 (13)	0.0193 (4)	
C46	0.5465 (3)	0.6782 (2)	0.40772 (14)	0.0246 (5)	
H46	0.5484	0.6222	0.3723	0.030*	
C45	0.6455 (3)	0.6851 (2)	0.46676 (15)	0.0301 (6)	
H45	0.7123	0.6345	0.4700	0.036*	
C44	0.6449 (3)	0.7672 (2)	0.52088 (14)	0.0288 (6)	
H44	0.7106	0.7720	0.5605	0.035*	
C43	0.5444 (3)	0.8422 (2)	0.51493 (14)	0.0273 (5)	
H43	0.5420	0.8974	0.5509	0.033*	
C42	0.4476 (3)	0.8346 (2)	0.45520 (13)	0.0221 (5)	
H42	0.3826	0.8864	0.4521	0.027*	
C47	0.1657 (3)	0.6579 (2)	0.34644 (13)	0.0222 (5)	
C52	0.1789 (4)	0.5770 (3)	0.38702 (19)	0.0391 (7)	
H52	0.2746	0.5698	0.4070	0.047*	
C51	0.0544 (4)	0.5066 (3)	0.3987 (2)	0.0519 (10)	
H51	0.0688	0.4537	0.4259	0.062*	
C50	−0.0903 (4)	0.5147 (3)	0.3703 (2)	0.0420 (8)	
H50	−0.1735	0.4681	0.3782	0.050*	
C49	−0.1084 (3)	0.5934 (2)	0.32995 (17)	0.0337 (6)	
H49	−0.2047	0.6004	0.3105	0.040*	
C48	0.0174 (3)	0.6627 (2)	0.31811 (15)	0.0278 (5)	
H48	0.0021	0.7144	0.2902	0.033*	
C35	0.2753 (3)	0.85771 (19)	0.31755 (13)	0.0193 (4)	
C40	0.3444 (3)	0.9160 (2)	0.27141 (14)	0.0230 (5)	
H40	0.4086	0.8824	0.2443	0.028*	
C39	0.3212 (3)	1.0221 (2)	0.26434 (14)	0.0267 (5)	
H39	0.3694	1.0576	0.2331	0.032*	
C38	0.2256 (3)	1.0743 (2)	0.30421 (14)	0.0259 (5)	
H38	0.2120	1.1457	0.3010	0.031*	
C37	0.1509 (3)	1.0188 (2)	0.34875 (14)	0.0248 (5)	
H37	0.0847	1.0522	0.3748	0.030*	
C36	0.1749 (3)	0.9126 (2)	0.35462 (13)	0.0216 (5)	
H36	0.1222	0.8765	0.3843	0.026*	
C16	0.8371 (3)	−0.2312 (2)	0.07097 (14)	0.0259 (5)	
C15	0.7281 (3)	−0.1629 (2)	0.06614 (14)	0.0269 (5)	
H15	0.6522	−0.1765	0.0296	0.032*	
C14	0.7332 (3)	−0.0734 (2)	0.11670 (14)	0.0238 (5)	
H14	0.6582	−0.0286	0.1150	0.029*	
C13	0.8507 (3)	−0.05156 (18)	0.16955 (12)	0.0187 (4)	
C18	0.9592 (3)	−0.1224 (2)	0.17430 (14)	0.0227 (5)	
H18	1.0359	−0.1090	0.2106	0.027*	
C17	0.9515 (3)	−0.2127 (2)	0.12477 (14)	0.0262 (5)	
H17	1.0226	−0.2604	0.1277	0.031*	
C7	0.7727 (3)	0.10914 (19)	0.34383 (12)	0.0196 (5)	
C12	0.7334 (3)	−0.0017 (2)	0.34617 (14)	0.0238 (5)	
H12	0.6857	−0.0485	0.3077	0.029*	
C11	0.7662 (3)	−0.0412 (2)	0.40643 (15)	0.0271 (6)	
H11	0.7405	−0.1147	0.4084	0.032*	
C10	0.8370 (3)	0.0288 (2)	0.46348 (14)	0.0294 (6)	
H10	0.8573	0.0020	0.5038	0.035*	
C9	0.8781 (3)	0.1384 (2)	0.46135 (14)	0.0290 (6)	
H9	0.9269	0.1846	0.4999	0.035*	
C8	0.8460 (3)	0.1788 (2)	0.40143 (13)	0.0244 (5)	
H8	0.8734	0.2522	0.3997	0.029*	
C5	0.6489 (3)	0.24523 (18)	0.28198 (12)	0.0179 (4)	
C4	0.5715 (3)	0.2802 (2)	0.33544 (13)	0.0230 (5)	
H4	0.5746	0.2467	0.3739	0.028*	
C3	0.4887 (3)	0.3664 (2)	0.33071 (14)	0.0259 (5)	
H3	0.4375	0.3923	0.3663	0.031*	
C2	0.4841 (3)	0.4128 (2)	0.27214 (15)	0.0262 (5)	
H2	0.4300	0.4705	0.2679	0.031*	
C1	0.5610 (3)	0.3723 (2)	0.22002 (14)	0.0242 (5)	
H1	0.5550	0.4026	0.1804	0.029*	
C6	0.7347 (3)	0.15343 (18)	0.28092 (12)	0.0176 (4)	
C22	0.8732 (3)	0.36272 (19)	0.12008 (14)	0.0227 (5)	
C23	0.9768 (3)	0.2910 (2)	0.14045 (15)	0.0256 (5)	
C19	0.9463 (3)	0.1923 (2)	0.09100 (14)	0.0239 (5)	
C20	0.8205 (3)	0.2007 (2)	0.04434 (14)	0.0255 (5)	
C21	0.7727 (3)	0.3067 (2)	0.06293 (14)	0.0234 (5)	
C25	0.7491 (4)	0.1177 (3)	−0.01532 (15)	0.0357 (7)	
H25A	0.7881	0.1360	−0.0557	0.054*	
H25B	0.6412	0.1167	−0.0202	0.054*	
H25C	0.7724	0.0473	−0.0083	0.054*	
C24	1.0441 (4)	0.1043 (2)	0.08814 (18)	0.0352 (7)	
H24A	0.9867	0.0378	0.0641	0.053*	
H24B	1.0788	0.0944	0.1334	0.053*	
H24C	1.1297	0.1241	0.0651	0.053*	
C28	1.1065 (4)	0.3190 (3)	0.19625 (18)	0.0380 (7)	
H28A	1.1949	0.3500	0.1788	0.057*	
H28B	1.1267	0.2544	0.2138	0.057*	
H28C	1.0808	0.3705	0.2319	0.057*	
C27	0.8724 (4)	0.4769 (2)	0.15301 (16)	0.0316 (6)	
H27A	0.9419	0.5251	0.1339	0.047*	
H27B	0.9025	0.4832	0.2009	0.047*	
H27C	0.7722	0.4958	0.1454	0.047*	
C26	0.6498 (4)	0.3519 (2)	0.02314 (16)	0.0341 (6)	
H26A	0.5749	0.3708	0.0519	0.051*	
H26B	0.6032	0.2981	−0.0145	0.051*	
H26C	0.6932	0.4154	0.0067	0.051*	
B1	0.3166 (3)	0.7375 (2)	0.33184 (14)	0.0192 (5)	
Cl1	0.49270 (7)	0.12986 (5)	0.10753 (3)	0.02555 (12)	
Cl2	0.82834 (11)	−0.34453 (6)	0.00791 (4)	0.04097 (18)	
N3	0.8735 (2)	0.04187 (16)	0.21931 (11)	0.0206 (4)	
H3N	0.912 (4)	0.033 (2)	0.2572 (18)	0.025 (8)*	
N2	0.7748 (2)	0.11793 (15)	0.22150 (10)	0.0168 (4)	
N1	0.6440 (2)	0.29113 (15)	0.22428 (11)	0.0186 (4)	
Ir1	0.747445 (11)	0.217327 (7)	0.145260 (5)	0.01615 (3)	

### Atomic displacement parameters (Å^2^)

**Table:**

	*U*^11^	*U*^22^	*U*^33^	*U*^12^	*U*^13^	*U*^23^
C29	0.0232 (12)	0.0170 (10)	0.0192 (11)	0.0060 (9)	0.0034 (9)	0.0039 (9)
C30	0.0266 (13)	0.0256 (12)	0.0213 (12)	0.0063 (10)	0.0005 (10)	0.0023 (10)
C31	0.0413 (16)	0.0323 (14)	0.0171 (12)	0.0103 (12)	−0.0026 (11)	−0.0014 (10)
C32	0.0482 (18)	0.0359 (14)	0.0183 (12)	0.0205 (13)	0.0107 (12)	0.0059 (11)
C33	0.0310 (14)	0.0336 (14)	0.0288 (14)	0.0140 (11)	0.0116 (12)	0.0111 (11)
C34	0.0241 (12)	0.0220 (11)	0.0241 (12)	0.0055 (9)	0.0041 (10)	0.0040 (10)
C41	0.0188 (11)	0.0202 (10)	0.0187 (11)	0.0011 (9)	0.0035 (9)	0.0028 (9)
C46	0.0271 (13)	0.0225 (11)	0.0233 (12)	0.0063 (10)	0.0008 (10)	−0.0003 (10)
C45	0.0313 (14)	0.0325 (14)	0.0278 (14)	0.0126 (11)	−0.0011 (12)	0.0057 (11)
C44	0.0298 (14)	0.0351 (14)	0.0205 (12)	0.0027 (11)	−0.0013 (11)	0.0059 (11)
C43	0.0312 (14)	0.0288 (13)	0.0207 (12)	0.0014 (11)	0.0063 (11)	0.0005 (10)
C42	0.0231 (12)	0.0232 (11)	0.0208 (12)	0.0035 (9)	0.0065 (10)	0.0033 (9)
C47	0.0208 (12)	0.0240 (11)	0.0211 (12)	0.0023 (9)	0.0043 (10)	0.0003 (9)
C52	0.0274 (15)	0.0381 (16)	0.053 (2)	−0.0004 (12)	0.0012 (14)	0.0204 (15)
C51	0.0419 (19)	0.0440 (18)	0.072 (3)	−0.0071 (15)	0.0017 (18)	0.0325 (19)
C50	0.0298 (16)	0.0382 (16)	0.056 (2)	−0.0090 (13)	0.0102 (15)	0.0091 (15)
C49	0.0197 (13)	0.0391 (15)	0.0388 (17)	−0.0009 (11)	0.0035 (12)	−0.0006 (13)
C48	0.0235 (13)	0.0316 (13)	0.0274 (14)	0.0031 (10)	0.0028 (11)	0.0029 (11)
C35	0.0168 (11)	0.0197 (10)	0.0202 (11)	0.0031 (8)	−0.0006 (9)	0.0008 (9)
C40	0.0205 (12)	0.0226 (11)	0.0262 (13)	0.0037 (9)	0.0042 (10)	0.0031 (10)
C39	0.0255 (13)	0.0274 (12)	0.0277 (13)	0.0010 (10)	0.0022 (11)	0.0097 (11)
C38	0.0279 (13)	0.0197 (11)	0.0289 (14)	0.0052 (10)	−0.0042 (11)	0.0031 (10)
C37	0.0248 (13)	0.0246 (12)	0.0238 (12)	0.0099 (10)	−0.0021 (10)	−0.0021 (10)
C36	0.0233 (12)	0.0243 (11)	0.0174 (11)	0.0067 (9)	0.0008 (10)	0.0018 (9)
C16	0.0366 (15)	0.0171 (11)	0.0228 (12)	−0.0013 (10)	0.0105 (11)	−0.0007 (9)
C15	0.0329 (14)	0.0243 (12)	0.0206 (12)	−0.0012 (10)	0.0005 (11)	0.0003 (10)
C14	0.0254 (13)	0.0204 (11)	0.0245 (13)	0.0018 (9)	0.0005 (10)	0.0023 (9)
C13	0.0226 (12)	0.0179 (10)	0.0165 (11)	0.0034 (9)	0.0049 (9)	0.0031 (8)
C18	0.0238 (12)	0.0215 (11)	0.0232 (12)	0.0056 (9)	0.0028 (10)	0.0033 (9)
C17	0.0312 (14)	0.0194 (11)	0.0303 (14)	0.0071 (10)	0.0113 (11)	0.0035 (10)
C7	0.0193 (11)	0.0239 (11)	0.0174 (11)	0.0072 (9)	0.0035 (9)	0.0051 (9)
C12	0.0219 (12)	0.0242 (12)	0.0254 (13)	0.0017 (9)	0.0007 (10)	0.0070 (10)
C11	0.0236 (13)	0.0308 (13)	0.0325 (14)	0.0064 (10)	0.0088 (11)	0.0176 (11)
C10	0.0283 (14)	0.0468 (16)	0.0194 (12)	0.0157 (12)	0.0069 (11)	0.0145 (12)
C9	0.0313 (14)	0.0397 (15)	0.0161 (12)	0.0117 (12)	0.0009 (11)	0.0007 (11)
C8	0.0290 (13)	0.0250 (12)	0.0203 (12)	0.0088 (10)	0.0035 (10)	0.0026 (10)
C5	0.0197 (11)	0.0158 (10)	0.0177 (11)	0.0020 (8)	0.0007 (9)	0.0024 (8)
C4	0.0252 (12)	0.0237 (11)	0.0214 (12)	0.0058 (10)	0.0044 (10)	0.0048 (9)
C3	0.0250 (13)	0.0247 (12)	0.0289 (14)	0.0081 (10)	0.0069 (11)	0.0005 (10)
C2	0.0240 (13)	0.0188 (11)	0.0354 (15)	0.0074 (9)	−0.0012 (11)	0.0025 (10)
C1	0.0285 (13)	0.0196 (11)	0.0255 (13)	0.0072 (10)	0.0010 (11)	0.0052 (10)
C6	0.0183 (11)	0.0167 (10)	0.0176 (11)	0.0018 (8)	0.0007 (9)	0.0034 (8)
C22	0.0199 (12)	0.0202 (11)	0.0272 (13)	−0.0014 (9)	0.0018 (10)	0.0056 (10)
C23	0.0208 (12)	0.0266 (12)	0.0300 (14)	−0.0004 (10)	0.0021 (11)	0.0105 (11)
C19	0.0249 (13)	0.0247 (12)	0.0238 (12)	0.0016 (10)	0.0097 (10)	0.0065 (10)
C20	0.0296 (14)	0.0264 (12)	0.0211 (12)	0.0002 (10)	0.0090 (11)	0.0046 (10)
C21	0.0219 (12)	0.0241 (11)	0.0242 (13)	−0.0018 (9)	0.0008 (10)	0.0095 (10)
C25	0.0418 (17)	0.0386 (15)	0.0216 (13)	−0.0050 (13)	0.0076 (12)	−0.0059 (11)
C24	0.0314 (15)	0.0312 (14)	0.0488 (19)	0.0087 (12)	0.0196 (14)	0.0114 (13)
C28	0.0284 (15)	0.0431 (17)	0.0401 (18)	−0.0023 (13)	−0.0091 (13)	0.0144 (14)
C27	0.0345 (15)	0.0212 (12)	0.0368 (16)	−0.0015 (11)	0.0012 (13)	0.0036 (11)
C26	0.0354 (16)	0.0361 (15)	0.0307 (15)	0.0014 (12)	−0.0068 (13)	0.0159 (12)
B1	0.0178 (12)	0.0194 (12)	0.0203 (13)	0.0044 (9)	0.0028 (10)	0.0013 (10)
Cl1	0.0191 (3)	0.0295 (3)	0.0263 (3)	−0.0005 (2)	−0.0014 (2)	0.0054 (2)
Cl2	0.0640 (5)	0.0239 (3)	0.0319 (4)	0.0004 (3)	0.0143 (4)	−0.0073 (3)
N3	0.0238 (11)	0.0211 (9)	0.0166 (10)	0.0089 (8)	−0.0023 (8)	0.0004 (8)
N2	0.0175 (9)	0.0165 (9)	0.0163 (9)	0.0038 (7)	0.0015 (8)	0.0019 (7)
N1	0.0184 (10)	0.0169 (9)	0.0206 (10)	0.0042 (7)	−0.0003 (8)	0.0033 (7)
Ir1	0.01740 (5)	0.01629 (4)	0.01465 (4)	0.00196 (3)	0.00082 (3)	0.00322 (3)

### Geometric parameters (Å, º)

**Table:**

C29—C30	1.408 (4)	C7—C8	1.395 (4)
C29—C34	1.408 (4)	C7—C12	1.401 (3)
C29—B1	1.642 (4)	C7—C6	1.477 (3)
C30—C31	1.397 (4)	C12—C11	1.390 (4)
C30—H30	0.9300	C12—H12	0.9300
C31—C32	1.383 (5)	C11—C10	1.382 (4)
C31—H31	0.9300	C11—H11	0.9300
C32—C33	1.386 (5)	C10—C9	1.389 (4)
C32—H32	0.9300	C10—H10	0.9300
C33—C34	1.392 (4)	C9—C8	1.388 (4)
C33—H33	0.9300	C9—H9	0.9300
C34—H34	0.9300	C8—H8	0.9300
C41—C42	1.399 (3)	C5—N1	1.365 (3)
C41—C46	1.413 (3)	C5—C4	1.385 (3)
C41—B1	1.650 (4)	C5—C6	1.462 (3)
C46—C45	1.392 (4)	C4—C3	1.396 (3)
C46—H46	0.9300	C4—H4	0.9300
C45—C44	1.388 (4)	C3—C2	1.384 (4)
C45—H45	0.9300	C3—H3	0.9300
C44—C43	1.390 (4)	C2—C1	1.382 (4)
C44—H44	0.9300	C2—H2	0.9300
C43—C42	1.391 (4)	C1—N1	1.342 (3)
C43—H43	0.9300	C1—H1	0.9300
C42—H42	0.9300	C6—N2	1.314 (3)
C47—C52	1.399 (4)	C22—C21	1.422 (4)
C47—C48	1.403 (4)	C22—C23	1.441 (3)
C47—B1	1.655 (4)	C22—C27	1.492 (4)
C52—C51	1.395 (4)	C22—Ir1	2.174 (2)
C52—H52	0.9300	C23—C19	1.451 (4)
C51—C50	1.384 (5)	C23—C28	1.499 (4)
C51—H51	0.9300	C23—Ir1	2.163 (3)
C50—C49	1.379 (5)	C19—C20	1.414 (4)
C50—H50	0.9300	C19—C24	1.492 (4)
C49—C48	1.397 (4)	C19—Ir1	2.213 (3)
C49—H49	0.9300	C20—C21	1.459 (4)
C48—H48	0.9300	C20—C25	1.500 (4)
C35—C40	1.405 (4)	C20—Ir1	2.182 (3)
C35—C36	1.408 (3)	C21—C26	1.510 (4)
C35—B1	1.655 (3)	C21—Ir1	2.142 (2)
C40—C39	1.399 (3)	C25—H25A	0.9600
C40—H40	0.9300	C25—H25B	0.9600
C39—C38	1.391 (4)	C25—H25C	0.9600
C39—H39	0.9300	C24—H24A	0.9600
C38—C37	1.385 (4)	C24—H24B	0.9600
C38—H38	0.9300	C24—H24C	0.9600
C37—C36	1.397 (3)	C28—H28A	0.9600
C37—H37	0.9300	C28—H28B	0.9600
C36—H36	0.9300	C28—H28C	0.9600
C16—C15	1.382 (4)	C27—H27A	0.9600
C16—C17	1.384 (4)	C27—H27B	0.9600
C16—Cl2	1.753 (3)	C27—H27C	0.9600
C15—C14	1.396 (4)	C26—H26A	0.9600
C15—H15	0.9300	C26—H26B	0.9600
C14—C13	1.388 (4)	C26—H26C	0.9600
C14—H14	0.9300	Cl1—Ir1	2.4024 (6)
C13—C18	1.402 (3)	N3—N2	1.380 (3)
C13—N3	1.407 (3)	N3—H3N	0.83 (3)
C18—C17	1.387 (4)	N2—Ir1	2.1233 (19)
C18—H18	0.9300	N1—Ir1	2.081 (2)
C17—H17	0.9300		
			
C30—C29—C34	115.0 (2)	C2—C3—H3	120.6
C30—C29—B1	122.0 (2)	C4—C3—H3	120.6
C34—C29—B1	122.7 (2)	C1—C2—C3	119.2 (2)
C31—C30—C29	122.6 (3)	C1—C2—H2	120.4
C31—C30—H30	118.7	C3—C2—H2	120.4
C29—C30—H30	118.7	N1—C1—C2	122.7 (2)
C32—C31—C30	120.2 (3)	N1—C1—H1	118.6
C32—C31—H31	119.9	C2—C1—H1	118.6
C30—C31—H31	119.9	N2—C6—C5	114.8 (2)
C31—C32—C33	119.3 (3)	N2—C6—C7	124.9 (2)
C31—C32—H32	120.4	C5—C6—C7	120.2 (2)
C33—C32—H32	120.4	C21—C22—C23	108.5 (2)
C32—C33—C34	119.9 (3)	C21—C22—C27	125.7 (2)
C32—C33—H33	120.1	C23—C22—C27	125.8 (2)
C34—C33—H33	120.1	C21—C22—Ir1	69.55 (14)
C33—C34—C29	123.0 (3)	C23—C22—Ir1	70.19 (14)
C33—C34—H34	118.5	C27—C22—Ir1	126.5 (2)
C29—C34—H34	118.5	C22—C23—C19	107.2 (2)
C42—C41—C46	115.0 (2)	C22—C23—C28	126.3 (3)
C42—C41—B1	122.8 (2)	C19—C23—C28	125.9 (3)
C46—C41—B1	122.0 (2)	C22—C23—Ir1	70.99 (14)
C45—C46—C41	122.7 (2)	C19—C23—Ir1	72.50 (15)
C45—C46—H46	118.7	C28—C23—Ir1	128.4 (2)
C41—C46—H46	118.7	C20—C19—C23	108.3 (2)
C44—C45—C46	120.2 (2)	C20—C19—C24	127.6 (3)
C44—C45—H45	119.9	C23—C19—C24	123.8 (3)
C46—C45—H45	119.9	C20—C19—Ir1	70.06 (15)
C45—C44—C43	119.0 (3)	C23—C19—Ir1	68.80 (14)
C45—C44—H44	120.5	C24—C19—Ir1	131.44 (18)
C43—C44—H44	120.5	C19—C20—C21	108.1 (2)
C44—C43—C42	119.9 (2)	C19—C20—C25	126.9 (3)
C44—C43—H43	120.0	C21—C20—C25	125.0 (3)
C42—C43—H43	120.0	C19—C20—Ir1	72.40 (15)
C43—C42—C41	123.3 (2)	C21—C20—Ir1	68.80 (14)
C43—C42—H42	118.4	C25—C20—Ir1	125.59 (19)
C41—C42—H42	118.4	C22—C21—C20	107.6 (2)
C52—C47—C48	114.8 (2)	C22—C21—C26	126.8 (2)
C52—C47—B1	121.4 (2)	C20—C21—C26	125.3 (3)
C48—C47—B1	123.7 (2)	C22—C21—Ir1	71.98 (14)
C51—C52—C47	122.8 (3)	C20—C21—Ir1	71.79 (14)
C51—C52—H52	118.6	C26—C21—Ir1	126.9 (2)
C47—C52—H52	118.6	C20—C25—H25A	109.5
C50—C51—C52	120.6 (3)	C20—C25—H25B	109.5
C50—C51—H51	119.7	H25A—C25—H25B	109.5
C52—C51—H51	119.7	C20—C25—H25C	109.5
C49—C50—C51	118.5 (3)	H25A—C25—H25C	109.5
C49—C50—H50	120.7	H25B—C25—H25C	109.5
C51—C50—H50	120.7	C19—C24—H24A	109.5
C50—C49—C48	120.3 (3)	C19—C24—H24B	109.5
C50—C49—H49	119.9	H24A—C24—H24B	109.5
C48—C49—H49	119.9	C19—C24—H24C	109.5
C49—C48—C47	123.0 (3)	H24A—C24—H24C	109.5
C49—C48—H48	118.5	H24B—C24—H24C	109.5
C47—C48—H48	118.5	C23—C28—H28A	109.5
C40—C35—C36	114.9 (2)	C23—C28—H28B	109.5
C40—C35—B1	123.2 (2)	H28A—C28—H28B	109.5
C36—C35—B1	121.8 (2)	C23—C28—H28C	109.5
C39—C40—C35	123.2 (2)	H28A—C28—H28C	109.5
C39—C40—H40	118.4	H28B—C28—H28C	109.5
C35—C40—H40	118.4	C22—C27—H27A	109.5
C38—C39—C40	119.7 (2)	C22—C27—H27B	109.5
C38—C39—H39	120.2	H27A—C27—H27B	109.5
C40—C39—H39	120.2	C22—C27—H27C	109.5
C37—C38—C39	119.2 (2)	H27A—C27—H27C	109.5
C37—C38—H38	120.4	H27B—C27—H27C	109.5
C39—C38—H38	120.4	C21—C26—H26A	109.5
C38—C37—C36	120.1 (2)	C21—C26—H26B	109.5
C38—C37—H37	120.0	H26A—C26—H26B	109.5
C36—C37—H37	120.0	C21—C26—H26C	109.5
C37—C36—C35	122.9 (2)	H26A—C26—H26C	109.5
C37—C36—H36	118.6	H26B—C26—H26C	109.5
C35—C36—H36	118.6	C29—B1—C41	111.8 (2)
C15—C16—C17	121.5 (2)	C29—B1—C35	106.48 (19)
C15—C16—Cl2	119.1 (2)	C41—B1—C35	109.75 (19)
C17—C16—Cl2	119.4 (2)	C29—B1—C47	109.7 (2)
C16—C15—C14	119.4 (3)	C41—B1—C47	107.3 (2)
C16—C15—H15	120.3	C35—B1—C47	111.8 (2)
C14—C15—H15	120.3	N2—N3—C13	122.2 (2)
C13—C14—C15	119.7 (2)	N2—N3—H3N	114 (2)
C13—C14—H14	120.2	C13—N3—H3N	115 (2)
C15—C14—H14	120.2	C6—N2—N3	117.3 (2)
C14—C13—C18	120.2 (2)	C6—N2—Ir1	116.71 (15)
C14—C13—N3	123.9 (2)	N3—N2—Ir1	122.48 (15)
C18—C13—N3	115.9 (2)	C1—N1—C5	118.2 (2)
C17—C18—C13	119.8 (2)	C1—N1—Ir1	124.82 (17)
C17—C18—H18	120.1	C5—N1—Ir1	116.59 (15)
C13—C18—H18	120.1	N1—Ir1—N2	76.15 (7)
C16—C17—C18	119.3 (2)	N1—Ir1—C21	115.49 (9)
C16—C17—H17	120.3	N2—Ir1—C21	165.88 (9)
C18—C17—H17	120.3	N1—Ir1—C23	114.56 (10)
C8—C7—C12	120.1 (2)	N2—Ir1—C23	103.16 (9)
C8—C7—C6	119.5 (2)	C21—Ir1—C23	65.35 (10)
C12—C7—C6	120.4 (2)	N1—Ir1—C22	98.28 (9)
C11—C12—C7	119.5 (3)	N2—Ir1—C22	135.84 (9)
C11—C12—H12	120.3	C21—Ir1—C22	38.47 (10)
C7—C12—H12	120.3	C23—Ir1—C22	38.82 (9)
C10—C11—C12	120.0 (3)	N1—Ir1—C20	154.47 (9)
C10—C11—H11	120.0	N2—Ir1—C20	129.39 (9)
C12—C11—H11	120.0	C21—Ir1—C20	39.41 (10)
C11—C10—C9	120.8 (2)	C23—Ir1—C20	64.63 (11)
C11—C10—H10	119.6	C22—Ir1—C20	64.50 (10)
C9—C10—H10	119.6	N1—Ir1—C19	152.64 (10)
C8—C9—C10	119.7 (3)	N2—Ir1—C19	101.40 (8)
C8—C9—H9	120.2	C21—Ir1—C19	64.54 (10)
C10—C9—H9	120.2	C23—Ir1—C19	38.70 (10)
C9—C8—C7	119.9 (2)	C22—Ir1—C19	64.10 (9)
C9—C8—H8	120.0	C20—Ir1—C19	37.54 (10)
C7—C8—H8	120.0	N1—Ir1—Cl1	82.17 (6)
N1—C5—C4	122.0 (2)	N2—Ir1—Cl1	92.12 (6)
N1—C5—C6	114.7 (2)	C21—Ir1—Cl1	97.28 (7)
C4—C5—C6	123.3 (2)	C23—Ir1—Cl1	159.44 (8)
C5—C4—C3	119.0 (2)	C22—Ir1—Cl1	131.07 (7)
C5—C4—H4	120.5	C20—Ir1—Cl1	95.06 (8)
C3—C4—H4	120.5	C19—Ir1—Cl1	125.16 (7)
C2—C3—C4	118.9 (2)		
			
C34—C29—C30—C31	0.0 (4)	C7—C6—N2—Ir1	−166.92 (19)
B1—C29—C30—C31	173.9 (2)	C13—N3—N2—C6	134.8 (2)
C29—C30—C31—C32	−0.6 (4)	C13—N3—N2—Ir1	−67.1 (3)
C30—C31—C32—C33	0.6 (4)	C2—C1—N1—C5	1.7 (4)
C31—C32—C33—C34	−0.1 (4)	C2—C1—N1—Ir1	174.5 (2)
C32—C33—C34—C29	−0.5 (4)	C4—C5—N1—C1	−0.1 (4)
C30—C29—C34—C33	0.5 (3)	C6—C5—N1—C1	176.7 (2)
B1—C29—C34—C33	−173.3 (2)	C4—C5—N1—Ir1	−173.49 (19)
C42—C41—C46—C45	0.0 (4)	C6—C5—N1—Ir1	3.3 (3)
B1—C41—C46—C45	−174.6 (3)	C1—N1—Ir1—N2	−170.9 (2)
C41—C46—C45—C44	0.5 (4)	C5—N1—Ir1—N2	1.97 (17)
C46—C45—C44—C43	−0.2 (4)	C1—N1—Ir1—C21	17.6 (2)
C45—C44—C43—C42	−0.5 (4)	C5—N1—Ir1—C21	−169.51 (17)
C44—C43—C42—C41	1.0 (4)	C1—N1—Ir1—C23	90.7 (2)
C46—C41—C42—C43	−0.8 (4)	C5—N1—Ir1—C23	−96.38 (19)
B1—C41—C42—C43	173.8 (2)	C1—N1—Ir1—C22	53.8 (2)
C48—C47—C52—C51	−0.2 (5)	C5—N1—Ir1—C22	−133.33 (18)
B1—C47—C52—C51	−178.2 (3)	C1—N1—Ir1—C20	8.6 (3)
C47—C52—C51—C50	−0.4 (6)	C5—N1—Ir1—C20	−178.5 (2)
C52—C51—C50—C49	0.3 (6)	C1—N1—Ir1—C19	101.0 (3)
C51—C50—C49—C48	0.3 (5)	C5—N1—Ir1—C19	−86.1 (2)
C50—C49—C48—C47	−0.9 (5)	C1—N1—Ir1—Cl1	−76.8 (2)
C52—C47—C48—C49	0.8 (4)	C5—N1—Ir1—Cl1	96.12 (17)
B1—C47—C48—C49	178.7 (3)	C6—N2—Ir1—N1	−7.72 (17)
C36—C35—C40—C39	2.6 (4)	N3—N2—Ir1—N1	−166.01 (19)
B1—C35—C40—C39	−173.3 (2)	C6—N2—Ir1—C21	139.0 (3)
C35—C40—C39—C38	−0.1 (4)	N3—N2—Ir1—C21	−19.2 (5)
C40—C39—C38—C37	−2.1 (4)	C6—N2—Ir1—C23	104.74 (19)
C39—C38—C37—C36	1.7 (4)	N3—N2—Ir1—C23	−53.55 (19)
C38—C37—C36—C35	1.0 (4)	C6—N2—Ir1—C22	79.9 (2)
C40—C35—C36—C37	−3.1 (4)	N3—N2—Ir1—C22	−78.4 (2)
B1—C35—C36—C37	172.8 (2)	C6—N2—Ir1—C20	172.56 (17)
C17—C16—C15—C14	−0.1 (4)	N3—N2—Ir1—C20	14.3 (2)
Cl2—C16—C15—C14	179.1 (2)	C6—N2—Ir1—C19	144.34 (18)
C16—C15—C14—C13	2.6 (4)	N3—N2—Ir1—C19	−13.94 (19)
C15—C14—C13—C18	−3.6 (4)	C6—N2—Ir1—Cl1	−89.12 (17)
C15—C14—C13—N3	173.9 (2)	N3—N2—Ir1—Cl1	112.59 (17)
C14—C13—C18—C17	2.1 (4)	C22—C21—Ir1—N1	69.91 (17)
N3—C13—C18—C17	−175.6 (2)	C20—C21—Ir1—N1	−173.88 (14)
C15—C16—C17—C18	−1.4 (4)	C26—C21—Ir1—N1	−53.0 (3)
Cl2—C16—C17—C18	179.4 (2)	C22—C21—Ir1—N2	−74.0 (4)
C13—C18—C17—C16	0.4 (4)	C20—C21—Ir1—N2	42.2 (4)
C8—C7—C12—C11	0.8 (4)	C26—C21—Ir1—N2	163.1 (3)
C6—C7—C12—C11	−178.1 (2)	C22—C21—Ir1—C23	−36.82 (15)
C7—C12—C11—C10	0.1 (4)	C20—C21—Ir1—C23	79.39 (17)
C12—C11—C10—C9	−0.9 (4)	C26—C21—Ir1—C23	−159.8 (3)
C11—C10—C9—C8	0.8 (4)	C20—C21—Ir1—C22	116.2 (2)
C10—C9—C8—C7	0.0 (4)	C26—C21—Ir1—C22	−122.9 (3)
C12—C7—C8—C9	−0.9 (4)	C22—C21—Ir1—C20	−116.2 (2)
C6—C7—C8—C9	178.0 (2)	C26—C21—Ir1—C20	120.9 (3)
N1—C5—C4—C3	−1.4 (4)	C22—C21—Ir1—C19	−79.72 (16)
C6—C5—C4—C3	−177.9 (2)	C20—C21—Ir1—C19	36.49 (16)
C5—C4—C3—C2	1.3 (4)	C26—C21—Ir1—C19	157.3 (3)
C4—C3—C2—C1	0.2 (4)	C22—C21—Ir1—Cl1	154.68 (14)
C3—C2—C1—N1	−1.8 (4)	C20—C21—Ir1—Cl1	−89.11 (15)
N1—C5—C6—N2	−9.9 (3)	C26—C21—Ir1—Cl1	31.7 (2)
C4—C5—C6—N2	166.9 (2)	C22—C23—Ir1—N1	−71.61 (17)
N1—C5—C6—C7	168.8 (2)	C19—C23—Ir1—N1	172.47 (13)
C4—C5—C6—C7	−14.4 (4)	C28—C23—Ir1—N1	50.1 (3)
C8—C7—C6—N2	125.5 (3)	C22—C23—Ir1—N2	−152.20 (15)
C12—C7—C6—N2	−55.6 (4)	C19—C23—Ir1—N2	91.88 (15)
C8—C7—C6—C5	−53.1 (3)	C28—C23—Ir1—N2	−30.5 (3)
C12—C7—C6—C5	125.8 (3)	C22—C23—Ir1—C21	36.50 (16)
C21—C22—C23—C19	4.7 (3)	C19—C23—Ir1—C21	−79.42 (16)
C27—C22—C23—C19	−174.8 (3)	C28—C23—Ir1—C21	158.2 (3)
Ir1—C22—C23—C19	63.91 (17)	C19—C23—Ir1—C22	−115.9 (2)
C21—C22—C23—C28	176.7 (3)	C28—C23—Ir1—C22	121.7 (3)
C27—C22—C23—C28	−2.8 (4)	C22—C23—Ir1—C20	80.18 (17)
Ir1—C22—C23—C28	−124.2 (3)	C19—C23—Ir1—C20	−35.74 (15)
C21—C22—C23—Ir1	−59.18 (18)	C28—C23—Ir1—C20	−158.1 (3)
C27—C22—C23—Ir1	121.3 (3)	C22—C23—Ir1—C19	115.9 (2)
C22—C23—C19—C20	−3.8 (3)	C28—C23—Ir1—C19	−122.4 (3)
C28—C23—C19—C20	−175.8 (3)	C22—C23—Ir1—Cl1	70.8 (3)
Ir1—C23—C19—C20	59.08 (18)	C19—C23—Ir1—Cl1	−45.1 (3)
C22—C23—C19—C24	170.6 (2)	C28—C23—Ir1—Cl1	−167.52 (18)
C28—C23—C19—C24	−1.4 (4)	C21—C22—Ir1—N1	−121.06 (15)
Ir1—C23—C19—C24	−126.5 (2)	C23—C22—Ir1—N1	119.29 (16)
C22—C23—C19—Ir1	−62.91 (17)	C27—C22—Ir1—N1	−1.1 (2)
C28—C23—C19—Ir1	125.1 (3)	C21—C22—Ir1—N2	160.33 (14)
C23—C19—C20—C21	1.5 (3)	C23—C22—Ir1—N2	40.7 (2)
C24—C19—C20—C21	−172.7 (2)	C27—C22—Ir1—N2	−79.7 (3)
Ir1—C19—C20—C21	59.81 (18)	C23—C22—Ir1—C21	−119.7 (2)
C23—C19—C20—C25	−180.0 (3)	C27—C22—Ir1—C21	119.9 (3)
C24—C19—C20—C25	5.8 (4)	C21—C22—Ir1—C23	119.7 (2)
Ir1—C19—C20—C25	−121.7 (3)	C27—C22—Ir1—C23	−120.4 (3)
C23—C19—C20—Ir1	−58.30 (18)	C21—C22—Ir1—C20	39.13 (16)
C24—C19—C20—Ir1	127.5 (3)	C23—C22—Ir1—C20	−80.53 (17)
C23—C22—C21—C20	−3.8 (3)	C27—C22—Ir1—C20	159.1 (3)
C27—C22—C21—C20	175.7 (3)	C21—C22—Ir1—C19	80.97 (17)
Ir1—C22—C21—C20	−63.39 (17)	C23—C22—Ir1—C19	−38.69 (16)
C23—C22—C21—C26	−177.4 (3)	C27—C22—Ir1—C19	−159.1 (3)
C27—C22—C21—C26	2.1 (5)	C21—C22—Ir1—Cl1	−34.25 (18)
Ir1—C22—C21—C26	123.0 (3)	C23—C22—Ir1—Cl1	−153.90 (13)
C23—C22—C21—Ir1	59.57 (18)	C27—C22—Ir1—Cl1	85.7 (2)
C27—C22—C21—Ir1	−120.9 (3)	C19—C20—Ir1—N1	131.1 (2)
C19—C20—C21—C22	1.4 (3)	C21—C20—Ir1—N1	12.9 (3)
C25—C20—C21—C22	−177.1 (3)	C25—C20—Ir1—N1	−105.7 (3)
Ir1—C20—C21—C22	63.51 (18)	C19—C20—Ir1—N2	−49.54 (19)
C19—C20—C21—C26	175.1 (3)	C21—C20—Ir1—N2	−167.75 (13)
C25—C20—C21—C26	−3.4 (4)	C25—C20—Ir1—N2	73.7 (3)
Ir1—C20—C21—C26	−122.8 (3)	C19—C20—Ir1—C21	118.2 (2)
C19—C20—C21—Ir1	−62.10 (18)	C25—C20—Ir1—C21	−118.6 (3)
C25—C20—C21—Ir1	119.4 (3)	C19—C20—Ir1—C23	36.83 (15)
C30—C29—B1—C41	150.7 (2)	C21—C20—Ir1—C23	−81.38 (16)
C34—C29—B1—C41	−35.9 (3)	C25—C20—Ir1—C23	160.0 (3)
C30—C29—B1—C35	−89.4 (3)	C19—C20—Ir1—C22	80.01 (16)
C34—C29—B1—C35	83.9 (3)	C21—C20—Ir1—C22	−38.20 (15)
C30—C29—B1—C47	31.8 (3)	C25—C20—Ir1—C22	−156.8 (3)
C34—C29—B1—C47	−154.8 (2)	C21—C20—Ir1—C19	−118.2 (2)
C42—C41—B1—C29	151.8 (2)	C25—C20—Ir1—C19	123.2 (3)
C46—C41—B1—C29	−34.0 (3)	C19—C20—Ir1—Cl1	−146.48 (14)
C42—C41—B1—C35	33.9 (3)	C21—C20—Ir1—Cl1	95.32 (14)
C46—C41—B1—C35	−151.9 (2)	C25—C20—Ir1—Cl1	−23.3 (3)
C42—C41—B1—C47	−87.8 (3)	C20—C19—Ir1—N1	−135.02 (19)
C46—C41—B1—C47	86.4 (3)	C23—C19—Ir1—N1	−15.0 (3)
C40—C35—B1—C29	−24.8 (3)	C24—C19—Ir1—N1	101.9 (3)
C36—C35—B1—C29	159.6 (2)	C20—C19—Ir1—N2	143.14 (14)
C40—C35—B1—C41	96.3 (3)	C23—C19—Ir1—N2	−96.88 (15)
C36—C35—B1—C41	−79.3 (3)	C24—C19—Ir1—N2	20.1 (3)
C40—C35—B1—C47	−144.7 (2)	C20—C19—Ir1—C21	−38.29 (15)
C36—C35—B1—C47	39.7 (3)	C23—C19—Ir1—C21	81.69 (16)
C52—C47—B1—C29	90.4 (3)	C24—C19—Ir1—C21	−161.3 (3)
C48—C47—B1—C29	−87.4 (3)	C20—C19—Ir1—C23	−120.0 (2)
C52—C47—B1—C41	−31.3 (3)	C24—C19—Ir1—C23	117.0 (3)
C48—C47—B1—C41	151.0 (2)	C20—C19—Ir1—C22	−81.17 (16)
C52—C47—B1—C35	−151.7 (3)	C23—C19—Ir1—C22	38.81 (15)
C48—C47—B1—C35	30.5 (3)	C24—C19—Ir1—C22	155.8 (3)
C14—C13—N3—N2	3.3 (4)	C23—C19—Ir1—C20	120.0 (2)
C18—C13—N3—N2	−179.1 (2)	C24—C19—Ir1—C20	−123.0 (3)
C5—C6—N2—N3	171.2 (2)	C20—C19—Ir1—Cl1	42.29 (16)
C7—C6—N2—N3	−7.5 (3)	C23—C19—Ir1—Cl1	162.27 (12)
C5—C6—N2—Ir1	11.8 (3)	C24—C19—Ir1—Cl1	−80.8 (3)

### Hydrogen-bond geometry (Å, º)

Cg1, Cg2, Cg3, Cg4 and Cg5 are the centroids of the C29–C34, C41–C46, C35–C40, C7–C12 and C19–C23 rings, respectively.

**Table:**

*D*—H···*A*	*D*—H	H···*A*	*D*···*A*	*D*—H···*A*
C14—H14···Cl1	0.93	2.64	3.553 (3)	168
C2—H2···*Cg*1	0.93	2.71	3.463 (3)	139
C11—H11···*Cg*2^i^	0.93	2.61	3.365 (3)	139
C18—H18···*Cg*3^ii^	0.93	2.60	3.518 (3)	169
C37—H37···*Cg*4^iii^	0.93	2.65	3.489 (3)	150
C16—Cl2···*Cg*5^iv^	1.75 (1)	3.58 (1)	4.359 (3)	105 (1)

Symmetry codes: (i) *x*, *y*−1, *z*; (ii) *x*+1, *y*−1, *z*; (iii) *x*−1, *y*+1, *z*; (iv) −*x*+2, −*y*, −*z*.
