# Supplementary figures and images for: Crystal structure of chlorido­(2-{[2-(4-chloro­phen­yl)hydrazin-1-yl­idene-κN 1](phen­yl)meth­yl}pyridine-κN)(η5-penta­methyl­cyclo­penta­dien­yl)iridium(III) tetra­phenyl­borate
Source: Acta Crystallogr E Crystallogr Commun. 2015 Feb 18;71(Pt 3):m65–6. doi: 10.1107/S2056989015003023 (PMC4350695; doi:10.1107/S2056989015003023)

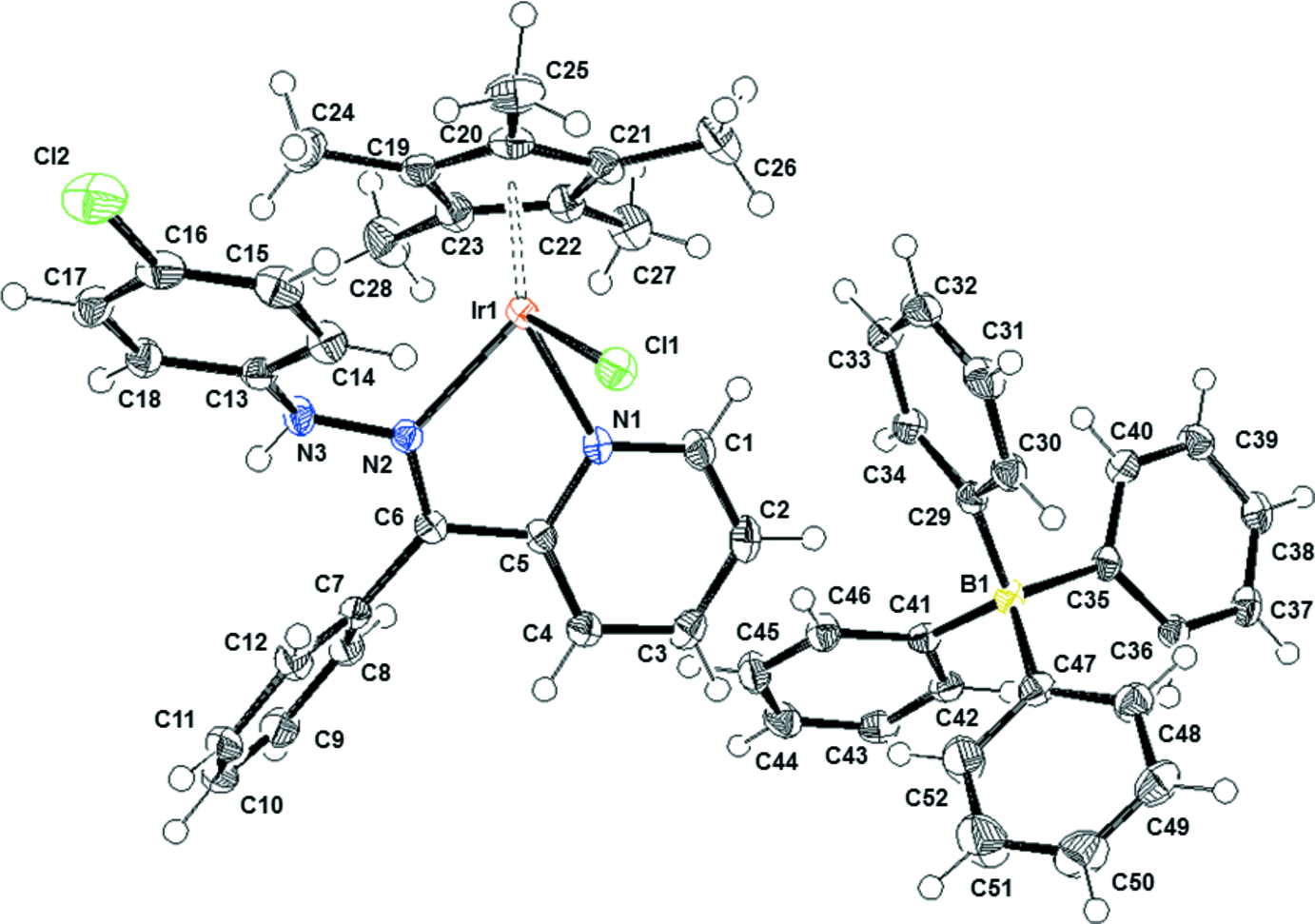

Supplement: Supplementary file 3 [file e-71-00m65-fig1.tif]

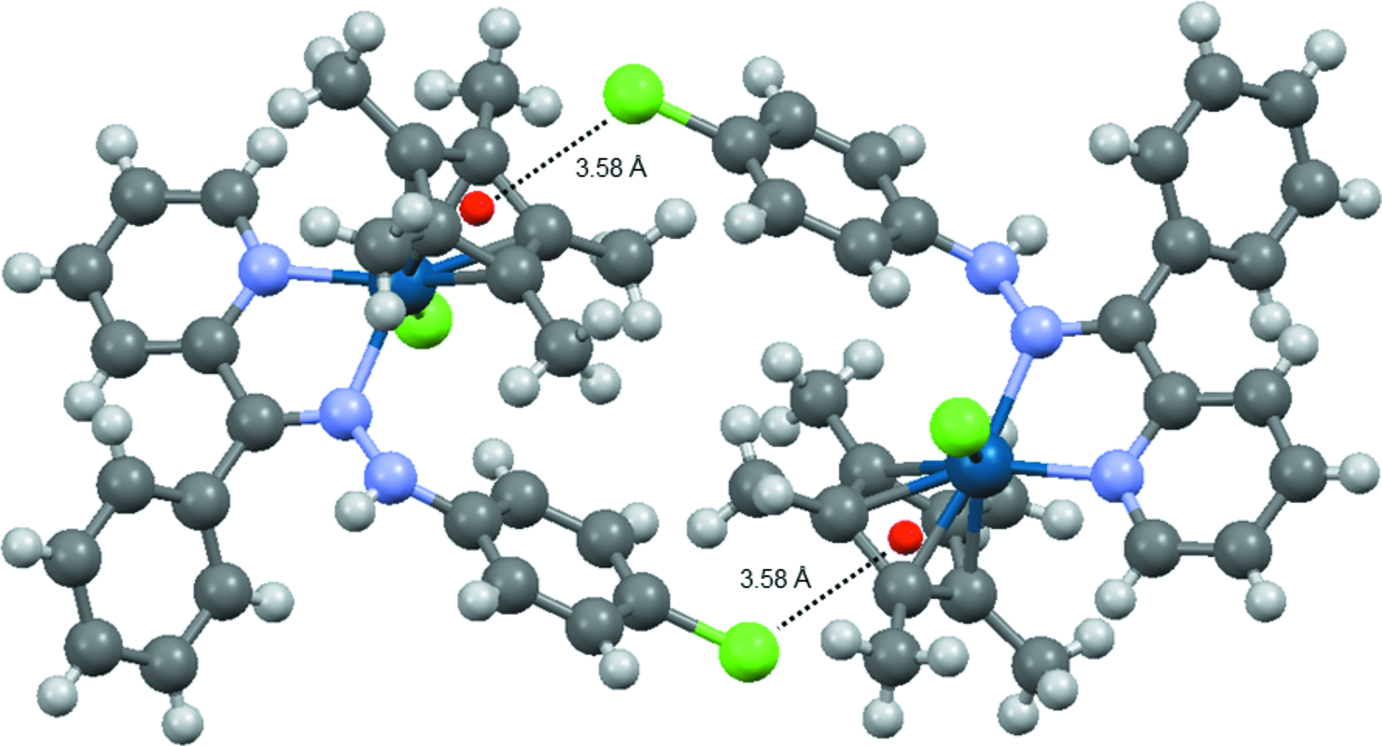

Supplement: Supplementary file 4 [file e-71-00m65-fig2.tif]
